# An Empirical Study on the Relationship between Stock Price Information and Enterprise Innovation Management Based on Information Learning Mechanism

**DOI:** 10.1155/2022/9425405

**Published:** 2022-05-17

**Authors:** Min Bai

**Affiliations:** Economics and Management School, Wuhan University, Wuhan 430072, China

## Abstract

The capital market economy had created a good environment for the development of enterprises, and innovation had brought great impetus to the sustainable development of enterprises. However, the research on the relationship between stock market and enterprise innovation was not deep enough. Therefore, this paper proposed an empirical study on the relationship between stock price information and enterprise innovation management based on information learning mechanism, in order to provide reference for enterprise innovation and development. Firstly, taking the A-share listed enterprises from 2005 to 2020 as the research sample, this paper analyzed the factors that may affect the innovation behavior of enterprises, such as enterprise stock price information, peer stock price information, and IPO, and put forward relevant assumptions. Secondly, according to various factors that restrict the innovation behavior of enterprises, this paper defined relevant indicators to reflect the change law of enterprise stock price information, peer stock price information, and IPO and described the restrictive relationship between exploratory innovation and developmental innovation with different influencing factors by constructing a correlation regression model. Finally, through empirical analysis, the results show that exploratory innovation and developmental innovation are not affected by the enterprise's own stock price information, but with the increase of peer stock price information, enterprise exploratory innovation becomes more sensitive to peer stock price information. Although the share price information of peers has no significant impact on enterprise development and innovation, after IPO, enterprises are more sensitive to the share price information of peers in exploratory innovation and developmental innovation. However, with the passage of time, the impact of peer stock price information on enterprise developmental innovation is gradually weakened.

## 1. Introduction

The enterprise development strategy driven by innovation is an important way for countries to realize economic transformation. Innovation can provide power for sustainable development for enterprises. With the rapid development of capital market economy, IPO has become an important way of financing for most enterprises [1]. The stock market mainly uses various resource allocation and R&D to reduce the risks in the process of enterprise innovation and thus promotes the development of the overall economy through technological innovation. Therefore, the stock market is an important source of financing for innovation activities, and the innovation and development of enterprises cannot be separated from the support of the capital market. However, in the context of the rapid development of the world economy, enterprises may face investment costs and unpredictable risks in the process of innovation and development. The relationship between the stock market and enterprise innovation has attracted extensive attention of relevant scholars.

Usually, enterprises are faced with huge investment costs and uncertain risks in the process of technological innovation. Capital market can provide services for enterprise innovation by effectively easing financing constraints and risk dispersion. Capital market is very important for the generation and aggregation of information. Information transmission is the internal mechanism of capital market promoting enterprise innovation and development. Effective information transmission can not only reduce information asymmetry, reduce capital cost, and promote enterprise innovation, but also help managers improve the efficiency of technological innovation decision-making and promote enterprises to obtain competitive advantage in the product market [2]. Investors can have different ways to obtain information and business philosophy by using the stock market. The stock price summarizes the information of all market participants through trading [3], and the change of stock price can reflect the information transmitted by the capital market. Compared with publicly disclosed information, stock price information comes from a wider range of sources. The stock price can not only reflect the results of the actual decision-making of the enterprise, but also provide guidance for the operation and management decision-making of the enterprise. Managers can improve the efficiency of investment decision-making and realize the effective allocation of resources through the learning of stock price information [4, 5].

As an important way of information transmission, stock price information has attracted extensive attention of scholars. Relevant research shows that enterprises pay more attention to the share price information of their peers, and learning information from the share price of their peers can help enterprises improve the efficiency of innovation activities [6]. However, there are few research results on the relationship between stock price information and enterprise independent innovation. Because innovation determines the sustainable development of enterprises, it is very important for enterprises to study the relationship between innovation and stock market on the basis of considering the important factor of stock price information. Therefore, in order to deeply explore the influencing factors of enterprise innovation and development, starting from the enterprise innovation and development strategy, this paper successively analyzed the factors that may affect enterprise innovation and development, such as enterprise stock price information, peer stock price information, and IPO, and put forward relevant assumptions. Based on the information learning mechanism, this paper studied the relationship between various factors and enterprise innovation and then explored the main factors affecting enterprise innovation through empirical analysis.

## 2. Related Works

Enterprise innovation can not only bring vitality to the survival and development of enterprises, but also an important driving force to promote economic growth. Researchers have proposed two different organizational learning behaviors in their early years: utilization and exploration [7]. On the one hand, enterprises should improve and upgrade on the basis of existing technical knowledge to meet the needs of the current market and customers, so as to increase the current income. On the other hand, enterprises need to actively explore new technical knowledge, reflect forward-looking market orientation, enhance long-term competitiveness, and increase future earnings. According to the intensity or degree of technological change in the process of innovation, technological innovation behavior usually includes exploratory innovation and developmental innovation, that is, dual intelligence innovation. There are both connections and essential differences between exploratory innovation and developmental innovation. In order to quickly adapt to the changes of the external environment and enhance the competitive advantage of the market, dual innovation is the basis of the sustainable development of enterprises. Facing the limited internal resource conditions and fierce external market competition, how enterprises make decisions on two different innovation behaviors has become a widespread concern of researchers in related fields.

Stock price information mainly refers to the private information contained in the stock price, which may be new information for managers and can provide decision-making reference for enterprise production management and investment management. People generally use price asynchrony to measure the private information in stock price. The asynchrony of stock prices reflects the changes in the company's stock return, which cannot be explained by market and industry activities. At the same time, stock price information has been proved to be the best index to reflect the dynamic changes of stock price information. Stock price information may contain bull and bear market news related to enterprise innovation strategy. In order to study the sensitivity of enterprise technological innovation to stock price information, enterprise innovation activities can be divided into exploratory innovation and developmental innovation. Compared with development innovation, exploratory innovation requires greater investment, longer cycle, and higher prospect uncertainty risk. Exploratory innovation can promote enterprises to obtain more lasting competitive advantage. In order to explore the importance of management learning in enterprise innovation activities, we can study the impact of future stock price asynchrony on enterprise innovation results and then compare the role of enterprise stock price information and peer stock price information in enterprise innovation.

From the existing empirical research results, it is found that the impact of enterprise's own stock price information on exploratory innovation and developmental innovation is not significant. Relevant studies show that the sensitivity of investment to stock price depends on the amount of incremental information related to management contained in stock price [8]. The motivation of enterprise managers to learn stock price information depends not only on the amount of information, but also on the correlation between stock price information and investment strategy. Compared with the stock price information of the enterprise itself, managers pay more attention to the stock price information of their peers when making innovation decisions. With the increase of peer stock price information, enterprise exploratory innovation becomes more sensitive to peer stock price information. However, the stock price information of peers has no significant impact on enterprise development innovation. After the IPO, the regulatory environment and product market competition have changed. IPO decision will not only affect the enterprise's technological innovation strategy [9], but also have a positive impact on the source of enterprise information acquisition. IPO can enable enterprises to obtain information from a wider range of channels. They can not only learn information from their peers' share prices, but also rely on their own share price information [10]. Therefore, enterprises after IPO are more sensitive to the share price information of their peers in terms of exploratory innovation and developmental innovation. In addition, the impact of peer stock price information on enterprise development innovation will generally weaken over time.

Relevant studies mainly analyze the factors driving enterprise innovation from multiple perspectives. Among them, the impact of stock price information on managers' investment decisions has been confirmed by scholars. However, the research on the impact of stock price information on enterprise innovation is still not deep enough. For example, there are few studies on the relationship between stock price information and two-way innovation strategy, and the impact mechanism of stock price information on enterprise two-way innovation is not clear enough. Therefore, based on the development of China's capital market, this paper mainly takes Shanghai and Shenzhen A-share listed enterprises as samples to study the impact of stock price information on enterprise innovation activities, so as to provide reference for further exploring the factors affecting enterprise innovation. Due to the heterogeneity of stock price information sources, listed companies can learn information not only from their own stock price, but also from the stock price information of their peers. This provides a basis for further exploring the different impact mechanisms of enterprise stock price information and peer stock price information on innovation activities. Due to the changing regulatory environment and market competition faced by enterprises after IPO, IPO decision not only affects the innovation strategy of enterprises, but also has a positive impact on the source of enterprise information acquisition. It is of great significance for enriching relevant theoretical and empirical research to carry out in-depth changes in the sensitivity of enterprise innovation to peer stock price information after IPO.

## 3. Relevant Theories and the Hypothesis of This Study

### 3.1. Relevant Theoretical Basis

#### 3.1.1. The Relationship between Enterprise Stock Price Information and Innovation

The service of capital market for enterprise innovation and development largely depends on the effectiveness of information transmission. Stock price information can reflect not only the comprehensive effect of factors such as the degree of information asymmetry and the level of internal and external governance of the company, but also an important index to measure the efficiency of market information transmission. In the research on the influencing factors of stock price information, some scholars found that when facing the uncertainty of economic policy, enterprises have the motivation to spread more information, which may lead to the decline of stock price [11]. High quality audit improves governance, reduces information asymmetry between insiders and investors, and enhances the impact of enterprise specific information on stock price [12]. Cross shareholding can promote price synchronization and reduce price delay through noise reduction process [13]. State owned property rights play an important role in stock price information. The price synchronization of state-owned enterprises is significantly greater than that of non-state-owned enterprises [14]. In addition, some scholars have studied the impact of stock price information on economic development. Stock prices may contain new information that business managers have not yet obtained. Enterprise managers can learn effective information from the stock price as the basis for decision-making of investment and financing activities and realize the effective allocation of resources. Managers learn the stock price information and take the learning results as the basis for enterprise investment decision-making. Research shows that stock price information has a strong positive impact on the sensitivity of enterprise investment and stock price. Stock price information can reduce management efficiency through learning mechanism and contract mechanism [5]. When stock price information is abundant, investment is more sensitive to stock price. Price information also determines the relationship between external financing and growth opportunities. Investment can promote the development of enterprises, and price information can provide reference for enhancing the future profitability of enterprises [7]. Managers generally carry out various innovation activities after learning the stock price information, and the amount of stock price information determines the innovation achievements of enterprises to a certain extent [12]. Stock price information mainly promotes enterprise innovation by reducing financing constraints, improving operation efficiency and reducing management costs.

Some scholars have also studied the relationship between investment and stock price information and its influencing factors. For example, cross listing enables enterprises to obtain accurate information about enterprise investment returns from the stock market, and the investment of cross listed companies is more sensitive to stock prices. The implementation of insider trading law not only has a great impact on stock price information, but also can increase the sensitivity of investment return [15]. Some scholars believe that when managers take stock price as a signal of enterprise growth, their ability to filter stock price noise will be limited [16]. As an important source of information, stock price information has attracted the attention of enterprise managers. Exploratory innovation and developmental innovation are important technological innovation activities of enterprises. When making decisions on innovation, managers fully consider all kinds of relevant information, which can improve the effectiveness of investment strategy. The higher the information content of stock price, the greater the possibility of enterprises' innovation activities to obtain effective information. Compared with developmental innovation, exploratory innovation is a more radical innovation behavior, mainly seeking new opportunities and possibilities. Therefore, exploratory innovation needs more motivation to learn stock price information.

#### 3.1.2. The Relationship between Peer Stock Price Information and Innovation

Considering the heterogeneity of stock price information sources, enterprise managers can learn information not only from their own stock prices, but also from the stock prices of their peers. The content and value of peer stock price information will also affect the innovation activities of enterprises. When peer enterprises have a larger market share, informed traders are more willing to trade peer stocks [17]. When the stock price information of peers is higher or their product demand is more relevant, the investment decision of enterprises is more sensitive to the stock price of peers. Private information production and trading in the secondary market reduce the market liquidity, thus increasing the impact of noise on price information [18]. According to the existing shared emotion hypothesis, there is a positive correlation between the wrong valuation of public enterprises and the investment of private peer enterprises. The overestimation of the open market increases the investment of private enterprises, which can have a certain impact on the investment of private enterprises by relaxing the financing constraints [19]. The relationship between enterprise innovation activities and peer stock price information is affected by the content and value of peer stock price information. Compared with exploratory innovation, developmental innovation is mainly the development and expansion of the existing technical knowledge of enterprises. Managers pay little attention to the stock price information of their peers when making pioneering innovation decisions.

#### 3.1.3. The Relationship between Stock Price Information, IPO, and Innovation

Although IPO can effectively alleviate the financing constraints faced by enterprises in the process of innovation, it also exposes a series of problems such as short-sighted incentives and disclosure requirements that restrict innovation. This trade-off is expected to form a unique innovation strategy model in listed enterprises. These enterprises may have experienced the loss of technology inventors and the decline in the productivity of the remaining inventors after IPO. Listing has changed the strategy of enterprises pursuing technological innovation. The equity dispersion, agency problems, and short-term pressure brought by IPO lead to the reduction of R&D investment. Compared with development innovation, exploratory innovation has a longer cycle and greater risk, and the level of exploratory innovation of listed enterprises is lower [10]. Listed enterprises are not only transparent to external investors, but also incentive measures generally focus on traditional projects. The investment cycle of listed enterprises is relatively short, and their patents rely more on existing knowledge, while the patent knowledge of private enterprises is broader and more exploratory [20]. Considering the impact of ownership change related to listing on the subsequent innovation strategy of enterprises, private enterprises carrying out developmental innovation are more likely to be listed, and these enterprises will continue to pursue developmental innovation after listing.

IPO decision not only affects the innovation behavior of enterprises, but also provides a platform for enterprise information sources. Private enterprises can learn more valuable things from the stock price information of listed peer enterprises. After listing, enterprises have more information resources. Listed companies can not only learn information from their peers' share prices, but also rely on their own share prices as a source of information. Therefore, IPO will affect the relationship between two-way innovation and peer share price information. In addition, due to the different intensity of industry competition, the efficiency of stock market price information may have different effects on innovation activities. After the initial public offering, with the intensification of competition, enterprises pay more and more attention to the share price information of their peers. Therefore, the learning mechanism of IPO can have a great impact on the stock price innovation of enterprises.

### 3.2. Research Hypothesis and Empirical Scheme

According to the current research progress on enterprise stock price information, peers' stock price information, and IPO, as well as the research results on the relationship between them and enterprise innovation, this paper puts forward the following research hypotheses.

**Hypothesis 1 .**

As the enterprise stock price information increases, the enterprise exploratory innovation becomes more sensitive to its own stock price information.

**Hypothesis 2 .**

As the enterprise stock price information increases, the enterprise exploitative innovation is not affected by its own stock price information.

**Hypothesis 3 .**

As the peers' stock price information increases, the enterprise exploratory innovation becomes more sensitive to the peers' stock price information.

**Hypothesis 4 .**

As the peers' stock price information increases, the enterprise exploitative innovation is not affected by the peers' stock price information.

**Hypothesis 5 .**

After the IPO, the enterprise exploratory innovation becomes more sensitive to the peers' stock price information.

**Hypothesis 6 .**

After the IPO, the enterprise exploitative innovation becomes more sensitive to the peers' stock price information.

**Figure 1:**
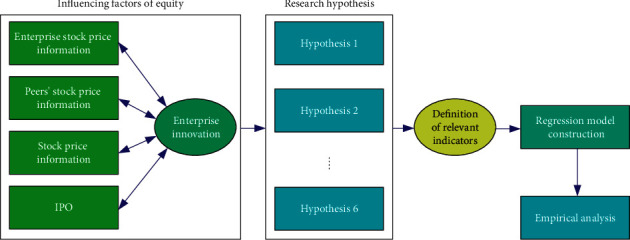
Empirical analysis and test scheme of related propositions.

In order to make a scientific evaluation of the above research proposition, this paper proposes to use the following scheme to conduct empirical analysis and test the relevant assumptions, as shown in Figure 1. Among them, for the proposed assumptions, we can define different indicators to describe the factors affecting the occurrence of enterprise innovation behavior and then build a regression model of enterprise innovation behavior to reflect the relationship between enterprise innovation and different factors.

## 4. Definition of Relevant Indicators and Model Construction

### 4.1. Definition of Related Indicators

In China, patents are classified into three types: invention, utility, and design. Patents that describe the degree and level of innovation of an enterprise are divided into two categories: invention patents and noninvention patents. Invention patents refer to new technical solutions for products and methods, emphasizing originality and novelty and having outstanding substantive features and significant progress compared with existing technologies. Therefore, invention patents largely reflect the ability and performance of an enterprise to engage in exploratory innovation. The more invention patents, the more new products or new technologies the company has that is different from its competitors. Noninvention patents include utility patents and design patents. A utility patent refers to a new technical solution suitable for practical use proposed for the shape, structure, or combination of the product. A design patent refers to a new design of the shape, pattern, color, or combination of the product that is rich in aesthetics and suitable for industrial application. Design and utility both involve the shape of a product. The shape involved in the utility is considered from the perspective of the technical effect and function of the product. However, the shape involved in the design is considered from the aesthetic point of view of the product. A noninvention patent requires progress in existing technology, and the creativity is significantly lower than that of an invention patent. To a large extent, noninvention patents describe the degree and level of an enterprise's exploitative innovation, that is, to what extent the enterprise has improved existing products or technologies.

Based on the existing research [21], this paper uses the number of patents granted each year to quantify the different innovation project decisions of enterprises. The exploratory innovation (*Explore*) of enterprises is measured by 1 plus the logarithm of the number of invention patents. In order to reflect the exploitative innovation (*Exploit*) of enterprises, this paper selects 1 plus the logarithm of design and utility patent counts.

For ease of description, this paper uses a dummy variable *IPO* to measure whether an enterprise is listed or not. In the observation year, if the enterprise has been listed, *IPO* = 1; otherwise it is 0.

Firstly, we need to calculate the price asynchrony, that is, the change of stock return that cannot be explained by market and industry factors, and take it as the main index to measure the stock price information. If the stock return of the enterprise is closely related to the market and industry return, the stock price of the enterprise is unlikely to convey the specific information of the enterprise, which plays an important reference role in the investment decision-making of the management. Because the data required for the calculation of this index is easy to collect and widely used, this paper adopts the existing methods to calculate the stock price information, which is denoted as *INF* [21]. The specific calculation formula is as follows:

(1) $$\begin{array}{c} INF_{i,t}=\beta_{0}+\beta_{m}INF_{m,t}+\beta_{j}INF_{j,t}+\varepsilon_{i,t}, \end{array}$$

where *INF*_*i*,*t*_ represents the weekly rate of return of enterprise *i* in period *t*, *INF*_*m*,*t*_ is the market return in period *t*, and *INF*_*j*,*t*_ is the return of industry *j* in which firm *i* is located. *β*_0_, *β*_*m*_, and *β*_*j*_ represent the weight coefficient, and *ε*_*i*,*t*_ denotes the adjustment coefficient.

Studies have found that price asynchrony can reflect more private information than noise [21]. Based on the existing research [14], the calculation model of peer stock price information can be expressed as

(2) $$\begin{array}{c} INF1_{i,t}=\ln\left(\frac{1-R_{i,t}^{2}}{R_{i,t}^{2}}\right), \end{array}$$

where *INF*1_*i*,*t*_ represents peer stock price information, and its calculation model reflects the weighted stock price information of circulation market value of all peer enterprises except *i* enterprise. If *R*_*i*,*t*_^2^ denotes the square of the regression coefficient, 1 − *R*_*i*,*t*_^2^ can be used to represent the asynchronous index of stock price, which is composed of two aspects: enterprise specific information and noise trading information.

In order to facilitate regression analysis, this paper uses relevant control variables to describe different characteristics that may affect enterprise innovation behavior, including relevant stock price information and its influencing factors. These control variables mainly include industrial competition (CO), industrial competition square (CO^2^), return on assets (Roa), enterprise scale (Size), enterprise capital structure(Lev), enterprise age(Age), etc. The selection and definition of control variables are shown in Table 1.

### 4.2. Model Construction

Since the information contained in stock price usually includes positive or negative investment signals, in order to analyze the impact of enterprise stock price information on enterprise two-way innovation activities, based on the existing research, this paper uses regression model to describe the change of enterprise innovation sensitivity to stock price information, and its calculation formula is as follows:

(3) $$\begin{array}{c} INN_{i,t+n}=\alpha_{t}+\eta_{i}+\beta_{1}INF_{i,t}+\beta_{2}INF_{i,t}^{2}+\gamma Cons_{i,t}+\varepsilon_{i,t}, \end{array}$$

where *i* is the firm and *t* denotes the year, *n*=1,2,3. *INN*_*i*,*t*+*n*_ represents the two measures for innovation activities: the exploratory innovation Explore_*i*,*t*+*n*_ and the exploitative innovation Exploit_*i*,*t*+*n*_. *INF*_*i*,*t*_ is the enterprise's own stock price information, *α*_*t*_ controls for year fixed effects, and *η*_*i*_ represents firm-fixed effects. *β*_1_, *β*_2_, and *γ* represent the weight coefficient, and *ε*_*i*,*t*_ denotes the adjustment coefficient. Cons_*i*,*t*_ is the control variables described in Table 1.

Considering the heterogeneity of stock price information sources, in order to analyze the impact of peer stock price information on enterprise innovation behavior, a regression model can be used to describe it, and its calculation formula is as follows:

(4) $$\begin{array}{c} INN_{i,t+n}=\alpha_{t}+\eta_{i}+\beta_{1}INF1_{i,t}+\beta_{2}INF1_{i,t}^{2}+\gamma Cons_{i,t}+\varepsilon_{i,t}, \end{array}$$

where *n*=1,2,3 and *INF*1_*i*,*t*_ is the peers' stock price information. The meanings of the remaining variables are as described above.

In addition, in order to analyze the impact of IPO on peer stock price information sensitivity in enterprise innovation behavior, the regression model can be used to describe as follows:

(5) $$\begin{array}{c} INN_{i,t+n}=\alpha_{t}+\eta_{i}+\beta_{1}IPO_{i,t}+\beta_{2}INF1_{i,t}+\beta_{3}{\text{IPO}}_{i,t}*INF1_{i,t}+\gamma {\text{Cons}}_{i,t}+\varepsilon_{i,t}, \end{array}$$

where *n*=1,2,3 and *IPO*_*i*,*t*_ is a dummy variable that equals one for the years that follow the IPO. The meanings of the variables are as described above.

## 5. Demonstration and Analysis

### 5.1. Sample Selection

**Figure 2:**
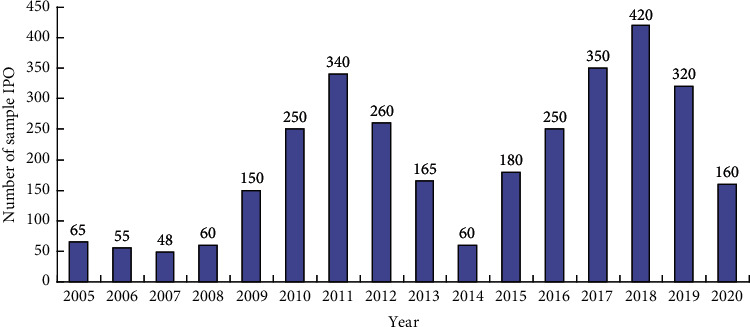
Time series of sample IPO activity.

In order to test the hypothesis proposed in this paper, this paper selects Chinese A-share listed enterprises from 2005 to 2020 as the research sample. According to the primary industry classification standard of Shenyin Wanguo Securities Research Institute, a total of 30 industries are adopted.  Figure 2 depicts the annual distribution of IPO of sample enterprises. In order to improve the effectiveness of empirical analysis and refer to the previous processing methods of relevant scholars, the corresponding enterprise data with the following characteristics are excluded: (1) transaction data of IPO year, (2) data of financial enterprises, and (3) data loss or abnormality. All data are from wind, CSMAR database, and China intellectual property network. Data preprocessing, descriptive statistics, and empirical analysis are all implemented in STATA15.0 software. All continuous variables are sorted in the 1st and 99th percentiles.

### 5.2. Data Description and Statistics

As shown in Table 2, the descriptive statistical results of different index parameters are reflected. According to the statistical results of the data in the table, the average value of exploratory innovation of the sample enterprises is 0.9167, the minimum value is 0, the maximum value is 3.8066, and the standard deviation is 0.8896, indicating that there are significant differences in exploratory innovation of different enterprises. Descriptive statistics for developmental innovation have similar results. The average value of enterprise stock price information is 0.2706, indicating that the enterprise specific information of Chinese listed enterprises is generally low.

**Figure 3:**
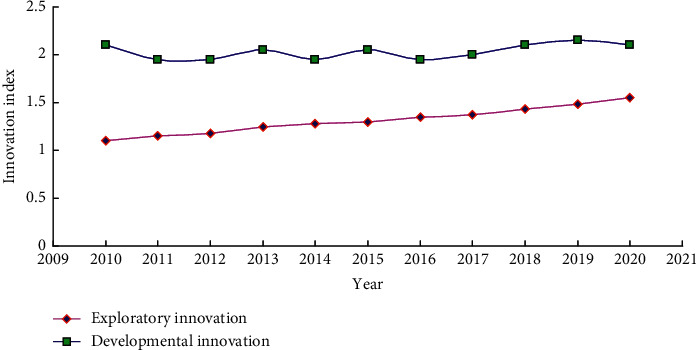
The change trend of exploratory innovation and developmental innovation of enterprises in the five years before and after IPO.

At the same time, we can get the change trend of exploratory innovation and developmental innovation of the sample enterprises in the five years before and after IPO from 2010 to 2020, as shown in Figure 3. Among them, exploratory innovation shows a trend of continuous enhancement, while the change of developmental innovation is relatively stable.

### 5.3. Regression Result Analysis

Through the regression simulation of the relationship between enterprise stock price information and innovation, the regression results of the impact of enterprise stock price information on innovation activities can be obtained, as shown in Table 3. According to the empirical results in Table 3, the stock price information of enterprises has no significant impact on exploratory innovation and developmental innovation. This shows that enterprises may not learn their own stock price information when formulating innovation strategies.

Through the regression simulation of the relationship between peer stock price information and innovation, the regression results of the impact of peer stock price information on enterprise innovation behavior can be obtained, as shown in Table 4. According to model (2) in Table 4, the coefficient of INF1 *∗* INF1 is 0.045, which is significant at the level of 5%. The results show that, with the increase of peer stock price information, exploratory innovation is more sensitive to peer stock price information. Models (4)–(6) represent the impact of peer stock price information on developmental innovation. However, compared with the stock price information of the enterprise itself, the stock price information of peers has no significant impact on the development and innovation of the enterprise. This result shows that there are some differences between exploratory innovation and developmental innovation under the influence of stock price information.

Private enterprises can rely on the share price of their peers as a source of information, especially when these enterprises are listed, and they can start to learn information from their own share price. Therefore, this will affect the relationship between the two-way innovation of enterprises and the share price information of their peers. Through the regression simulation of the relationship between stock price information, IPO, and enterprise innovation, the regression results of the impact of stock price information and IPO on enterprise innovation behavior can be obtained, as shown in Table 5. Models (1)–(3) in Table 5 show the impact of the interaction term INF1 *∗* IPO on enterprise exploratory innovation. This can be seen from the information coefficient of the IPO model (*P* + 0.053) that is 119.5 on the stock price of enterprises. When IPO = 0, *P* = -0.053; when IPO = 1, the influence coefficient is *p* = 0.066. In other words, enterprises after listing are more sensitive to the share price information of their peers in terms of exploratory innovation. Models (4)–(6) in Table 5 report the impact of the interaction term INF1 *∗* IPO on enterprise developmental innovation. In model (4) in Table 5, the influence coefficient of peer stock price information on enterprise development innovation is *p* = −0.086 + 0.124 *∗* IPO. The results show that, after IPO, the sensitivity of enterprise utilization innovation to peer stock price information is enhanced. With the passage of time, the impact of peer stock price information on enterprise development innovation is gradually weakened. This shows that the regression results are consistent with the hypothesis.

### 5.4. Robustness Test

In order to show the accuracy of the conclusion, the text tests the robustness of the above empirical results. Different industry classification standards may have a certain impact on the relevant variables of the industry index. For example, peer stock price information and industry competition indicators are closely related to the industry classification standards. In the robustness test, this paper uses wind secondary industry classification standards to remeasure the industry competition, peer stock price information, and other relevant indicators. In addition, considering the heterogeneity of demand for technological innovation in different industries, this paper selects high-tech industrial enterprises as the research sample to test the robustness of this research hypothesis. According to the classification of high-tech industries (manufacturing industry) (2017) implemented by the National Bureau of Statistics, this paper uses eight industries in the primary industry classification of ShenyinWanguo Securities Research Institute to represent high-tech industries, including electrical equipment, electronics, national defense and military industry, chemistry, mechanical equipment, computer, communication, pharmaceutical, and biological industry. Through the robustness test, the test results are highly consistent.

## 6. Conclusion

Because technological innovation can provide competitive advantages in the product market for the sustainable development of enterprises, this paper analyzed the important impact of stock price information on the two-way innovation behavior of enterprises. Firstly, by analyzing the development status of China's capital market, this paper explored the impact of enterprises' own stock price information on two-way innovation activities. The empirical results showed that enterprises' exploratory innovation and developmental innovation were not affected by their own stock price information. Secondly, the impact of stock price information on enterprise innovation behavior depended on the source of information. With the increase of peer stock price information, enterprise exploratory innovation became more sensitive to peer stock price information. However, the development innovation of enterprises was not affected by the stock price information of peers. The results showed that enterprises pay more attention to the stock price information of their peers when making exploratory innovation decisions. In addition, it explored the impact of IPO on the relationship between two-way innovation and stock price information. After IPO, enterprises had a wider range of information sources, but also faced changes in the product market competition environment. Enterprises' exploratory innovation and development innovation were more sensitive to the stock price information of their peers. With the passage of time, the impact of peer stock price information on enterprise development innovation was gradually weakened.

This study fully showed that enterprises should strengthen managers' awareness of learning stock price information and improve the efficiency of innovation investment. Since the function of optimizing the allocation of resources in the stock market depended on the effectiveness of information transmission, relevant departments should further improve the construction of information transmission mechanism in the capital market and improve the efficiency of capital market serving enterprise innovation. This study had certain reference significance for promoting enterprises to obtain innovative competitive advantage for sustainable development.

## Figures and Tables

**Table 1:** Definitions of variables.

Symbol		Variables	Variables definition
Dependent variables	Explore	Exploratory innovation	Logarithm of one plus invention patents
	Exploit	Exploitative innovation	Logarithm of one plus design and utility patents
Independent variables	IPO	Listed or not	IPO = 1 if the enterprise is listed
	INF	Enterprise stock price information	Calculate based on formula (1)
	INF1	Peers' stock price information	Weighted by circulating market value based on the industry classification standards
Control variables	CO	Industry competition	HHI index based on annual sales
	CO^2^	CO squared	HHI squared
	Roa	Return on assets	The ratio of net profit to total assets
	Size	Enterprise scale	Logarithm of total assets
	Lev	Leverage	Ratio of total liabilities to total assets
	Age	Enterprise age	Logarithm of the years from the establishment to the sample period

**Table 2:** Description and statistical results of different variables.

Indicators	Obs.	Mean	Std. dev.	Min	Max
Explore	6999	0.9167	0.8896	0.0000	3.8066
Exploit	6999	1.6409	1.2065	0.0000	4.8752
INF	7129	0.2706	0.8982	−1.7106	3.0247
INF1	13685	-0.5776	0.6168	−2.8291	1.0877
CO	17149	12.0530	15.8992	1.1707	77.5735
CO^2^	17149	3.9805	10.3236	0.0137	60.1764
Size	13552	4.7909	1.2532	2.1025	8.3894
Lev	13534	38.8531	18.4889	5.0482	81.1700
Roa	13141	10.0002	7.5961	0.3566	39.0863
Age	17149	2.4528	0.4937	0.8524	3.3711

**Table 3:** The restrictive relationship between enterprise stock price information and innovation.

Indicators	(1)	(2)	(3)	(4)	(5)	(6)
	Explore_t+1_	Explore_t+2_	Explore_t+3_	Exploit_t+1_	Exploit_t+2_	Exploit_t+3_
INF	−0.018	0.013	0.014	−0.023	0.008	−0.022
	(−0.883)	(0.529)	(0.496)	(−0.969)	(0.296)	(−0.663)
INF*∗*INF	−0.013	−0.015	−0.004	0.017	0.001	−0.002
	(−1.236)	(−1.173)	(−0.274)	(1.571)	(0.106)	(−0.096)
CO	−0.004	−0.013	−0.014	0.015	0.020	0.039^*∗*^
	(−0.367)	(−0.669)	(−0.813)	(0.852)	(0.986)	(1.868)
CO^2^	−0.004	0.027	0.001	−0.022	−0.013	−0.038
	(−0.225)	(0.654)	(0.038)	(−0.582)	(−0.345)	(−1.341)
Size	0.151^*∗∗*^	0.235^*∗∗∗*^	0.196^*∗*^	0.224^*∗∗∗*^	0.117	0.114
	(2.522)	(3.242)	(1.849)	(3.185)	(1.482)	(1.008)
Lev	0.002	−0.002	0.003	−0.002	0.001	0.001
	(1.065)	(−0.956)	(0.799)	(−0.948)	(0.508)	(0.415)
Roa	0.000	−0.001	0.003	0.018^*∗∗∗*^	0.023^*∗∗∗*^	0.024^*∗∗∗*^
	(0.066)	(−0.233)	(0.442)	(2.623)	(3.248)	(2.613)
Age	0.575^*∗*^	0.413	0.357	0.056	−0.089	0.239
	(1.656)	(0.822)	(0.592)	(0.141)	(−0.184)	(0.452)
Cons	−1.216	−0.831	−0.450	0.291	0.768	−0.039
	(−1.539)	(−0.743)	(−0.320)	(0.305)	(0.701)	(−0.033)

*Note*. ^*∗*^, ^*∗∗*^, and ^*∗∗∗*^, respectively, represent significance at the levels of 10%, 5%, and 1%.

**Table 4:** The restrictive relationship between peer stock price information and enterprise innovation.

Indicators	(1)	(2)	(3)	(4)	(5)	(6)
	Explore_t+1_	Explore_t+2_	Explore_t+3_	Exploit_t+1_	Exploit_t+2_	Exploit_t+3_
INF1	−0.054	0.064	0.033	0.010	0.118^*∗∗*^	−0.006
	(−1.345)	(1.475)	(0.677)	(0.186)	(2.132)	(−0.101)
INF1*∗*INF1	0.009	0.045^*∗∗*^	0.035^*∗*^	0.026	0.021	−0.014
	(0.581)	(2.492)	(1.855)	(1.114)	(0.938)	(−0.555)
CO	−0.005	0.003	0.008	0.003	0.005	0.014
	(−0.579)	(0.390)	(0.936)	(0.295)	(0.451)	(1.178)
CO^2^	0.005	−0.019^*∗*^	−0.033^*∗∗∗*^	−0.017	−0.009	−0.022
	(0.365)	(−1.663)	(−2.650)	(−0.814)	(−0.511)	(−1.222)
Size	0.117^*∗∗∗*^	0.090^*∗∗*^	0.089^*∗*^	0.206^*∗∗∗*^	0.194^*∗∗∗*^	0.148^*∗∗*^
	(2.797)	(2.043)	(1.720)	(3.855)	(3.698)	(2.380)
Lev	0.002^*∗*^	−0.002	−0.002	−0.002	0.002	0.002
	(1.653)	(−1.439)	(−1.229)	(−1.366)	(1.121)	(1.408)
Roa	0.000	0.005^*∗*^	0.005^*∗*^	0.012^*∗∗∗*^	0.011^*∗∗∗*^	0.005
	(0.085)	(1.951)	(1.737)	(3.236)	(3.220)	(1.276)
Age	0.230	0.520^*∗∗*^	0.478^*∗∗*^	−0.130	0.199	0.091
	(1.231)	(2.492)	(2.209)	(−0.481)	(0.739)	(0.351)
Cons	−0.586	−0.689	−0.391	1.176^*∗*^	0.361	0.792
	(−1.351)	(−1.426)	(−0.757)	(1.833)	(0.559)	(1.273)

*Note*. ^*∗*^, ^*∗∗*^, and ^*∗∗∗*^, respectively, represent significance at the levels of 10%, 5%, and 1%.

**Table 5:** The restrictive relationship between stock price information and IPO on enterprise innovation.

<!Col Count:7>Indicators	(1)	(2)	(3)	(4)	(5)	(6)
	Explore_t+1_	Explore_t+2_	Explore_t+3_	Exploit_t+1_	Exploit_t+2_	Exploit_t+3_
INF1	−0.046	−0.053	−0.062	−0.086	0.056	0.052
	(−1.192)	(−1.330)	(−1.465)	(−1.597)	(1.129)	(1.064)
IPO	−0.017	−0.165^*∗∗∗*^	−0.071	−0.217^*∗∗∗*^	0.030	0.018
	(−0.276)	(−2.680)	(−0.967)	(−2.716)	(0.432)	(0.226)
INF1*∗*IPO	−0.052	0.119^*∗∗*^	0.109^*∗∗*^	0.124^*∗∗*^	0.075	−0.130^*∗∗*^
	(−1.156)	(2.519)	(1.994)	(1.997)	(1.337)	(−2.101)
CO	−0.005	0.002	0.007	0.004	0.005	0.013
	(−0.633)	(0.295)	(0.839)	(0.352)	(0.465)	(1.121)
CO^2^	0.007	−0.020^*∗*^	−0.034^*∗∗∗*^	−0.020	−0.010	−0.018
	(0.509)	(−1.742)	(−2.681)	(−0.977)	(−0.588)	(−1.051)
Size	0.120^*∗∗∗*^	0.136^*∗∗∗*^	0.115^*∗∗*^	0.261^*∗∗∗*^	0.192^*∗∗∗*^	0.135^*∗∗*^
	(2.588)	(2.842)	(2.001)	(4.463)	(3.497)	(2.102)
Lev	0.002	−0.004^*∗∗∗*^	−0.003^*∗*^	−0.005^*∗∗∗*^	0.002	0.003
	(1.299)	(−2.644)	(−1.695)	(−2.628)	(1.112)	(1.588)
Roa	−0.000	0.002	0.004	0.009^*∗∗*^	0.012^*∗∗∗*^	0.005
	(−0.014)	(0.830)	(1.360)	(2.179)	(3.137)	(1.194)
Age	0.239	0.442^*∗∗*^	0.436^*∗∗*^	−0.195	0.195	0.117
	(1.267)	(2.102)	(2.000)	(−0.721)	(0.719)	(0.445)
Cons	−0.595	−0.589	−0.356	1.245^*∗*^	0.347	0.770
	(−1.370)	(−1.214)	(−0.691)	(1.946)	(0.536)	(1.227)

*Note*. ^*∗*^, ^*∗∗*^, and ^*∗∗∗*^, respectively, represent significance at the levels of 10%, 5%, and 1%.

## Data Availability

The labeled dataset used to support the findings of this study is available from the author upon request.

## References

[1] Lennerts S., Schulze A., Tomczak T. (2020). The asymmetric effects of exploitation and exploration on radical and incremental innovation performance: an uneven affair. *European Management Journal*.

[2] Zhang Y., Zhang X. (2020). Patent growth and the long-run performance of VC-backed IPOs. *International Review of Economics & Finance*.

[3] Chen Q., Goldstein I., Jiang W. (2007). Price informativeness and investment sensitivity to stock price. *Review of Financial Studies*.

[4] Ferreira D., Manso G., Silva A. C. (2014). Incentives to innovate and the decision to go public or private. *Review of Financial Studies*.

[5] Xu L. (2021). Stock price informativeness and managerial inefficiency. *International Review of Economics & Finance*.

[6] Badertscher B. A., Shanthikumar D. M., Teoh S. H. (2019). Private firm investment and public peer misvaluation. *The Accounting Review*.

[7] March J. G. (1991). Exploration and exploitation in organizational learning. *Organization Science*.

[8] da Silva P. P. (2021). Do managers pay attention to the market? A review of the relationship between stock price informativeness and investment. *Journal of Multinational Financial Management*.

[9] Aggarwal V. A., Hsu D. H. (2014). Entrepreneurial exits and innovation. *Management Science*.

[10] Wies S., Moorman C. (2015). Going public: How stock market listing changes firm innovation behavior. *Journal of Marketing Research*.

[11] Shen H., Liu R., Xiong H., Hou F., Tang X. (2021). Economic policy uncertainty and stock price synchronicity: Evidence from China. *Pacific-Basin Finance Journal*.

[12] Almaharmeh M. I., Shehadeh A. A., Iskandrani M., Saleh M. H. (2021). Audit quality and stock price synchronicity: Evidence from emerging stock markets. *Journal of Asian Finance, Economics and Business*.

[13] Wen F., Yuan Y., Zhou W. X. (2021). Cross‐shareholding networks and stock price synchronicity: Evidence from China. *International Journal of Finance & Economics*.

[14] Goodell J. W., Li M., Liu D. (2021). Price informativeness and state-owned enterprises: Considering their heterogeneity. *International Review of Financial Analysis*.

[15] Edmans A., Jayaraman S., Schneemeier J. (2017). The source of information in prices and investment-price sensitivity. *Journal of Financial Economics*.

[16] Dessaint O., Foucault T., Frésard L., Matray A. (2019). Noisy stock prices and corporate investment. *Review of Financial Studies*.

[17] Bade M. (2016). Learning from peers’ prices and corporate investment under the influence of shocks. *Journal of Finance and Economics*.

[18] Gao H., Hsu P.-H., Li K. (2017). Innovation strategy of private firms. *Journal of Financial and Quantitative Analysis*.

[19] Defond M. L., Hung M. (2004). Investor protection and corporate governance: Evidence from worldwide CEO turnover. *Journal of Accounting Research*.

[20] Durnev A., Morck R., Yeung B., Zarowin P. (2003). Does greater firm-specific return variation mean more or less informed stock pricing?. *Journal of Accounting Research*.

[21] Baranchuk N., Kieschnick R., Moussawi R. (2014). Motivating innovation in newly public firms. *Journal of Financial Economics*.

